# Informed palliative care in nursing homes through the interRAI Palliative Care instrument: a study protocol based on the Medical Research Council framework

**DOI:** 10.1186/1471-2318-14-132

**Published:** 2014-12-05

**Authors:** Kirsten Hermans, Nele Spruytte, Joachim Cohen, Chantal Van Audenhove, Anja Declercq

**Affiliations:** Center for Care Research and Consultancy, KU Leuven – University of Leuven, LUCAS, Kapucijnenvoer 39/5310, B-3000 Leuven, Belgium; End-of-Life Care Research Group, Vrije Universiteit Brussel (VUB) & Ghent University, Laarbeeklaan 103, B-1090 Brussels, Belgium

**Keywords:** Palliative care, Nursing homes, Comprehensive geriatric assessment, interRAI Palliative Care instrument, Older adults, Study protocol

## Abstract

**Background:**

Nursing homes are important locations for palliative care. Through comprehensive geriatric assessments (CGAs), evaluations can be made of palliative care needs of nursing home residents. The interRAI Palliative Care instrument (interRAI PC) is a CGA that evaluates diverse palliative care needs of adults in all healthcare settings. The evaluation results in Client Assessment Protocols (CAPs: indications of problems that need addressing) and Scales (e.g. Palliative Index for Mortality (PIM)) which can be used to design, evaluate and adjust care plans. This study aims to examine the effect of using the interRAI PC on the quality of palliative care in nursing homes. Additionally, it aims to evaluate the feasibility and validity of the interRAI PC.

**Methods:**

This study covers phases 0, I and II of the Medical Research Council (MRC) framework for designing and evaluating complex interventions, with a longitudinal, quasi-experimental pretest-posttest design and with mixed methods of evaluation. In phase 0, a systematic literature search is conducted. In phase I, the interRAI PC is adapted for use in Belgium and implemented on the BelRAI-website and a practical training is developed. In phase II, the intervention is tested in fifteen nursing homes. Participating nursing homes fill out the interRAI PC during one year for all residents receiving palliative care. Using a pretest-posttest design with quasi-random assignment to the intervention or control group, the effect of the interRAI PC on the quality of palliative care is evaluated with the Palliative care Outcome Scale (POS). Psychometric analysis is conducted to evaluate the predictive validity of the PIM and the convergent validity of the CAP ‘Mood’ of the interRAI PC. Qualitative data regarding the usability and face validity of the instrument are collected through focus groups, interviews and field notes.

**Discussion:**

This is the first study to evaluate the validity and effect of the interRAI PC in nursing homes, following a methodology based on the MRC framework. This approach improves the study design and implementation and will contribute to a higher generalizability of results. The final result will be a psychometrically evaluated CGA for nursing home residents receiving palliative care.

**Trial registration:**

ClinicalTrials.gov NCT02281032. Registered October 30th, 2014.

## Background

The population of frail older people dying in nursing homes is rising [1–3]. In Belgium, the proportion of hospital deaths in people aged 65 and older decreased from 54.3 to 50.5 percent and care home deaths increased from 25.4 to 30.3 percent between 1998 and 2007 [3]. In the United States, 25 percent of the population aged 65 and over dies in a care home [4]. This percentage is expected to double by 2020 [4, 5]. Subsequently, nursing homes are playing an increasing role in caring for older adults with palliative care needs [1, 2].

Optimal palliative care in long-term care facilities requires a comprehensive evaluation of the different care needs of nursing home residents [6, 7]. A comprehensive geriatric assessment (CGA) is defined as ‘a multidimensional, interdisciplinary diagnostic process to determine the medical, psychological and functional capabilities of a frail older person in order to develop a coordinated and integrated plan for treatment and long-term follow-up’ [8]. The main objectives of a CGA are to improve diagnostic accuracy and treatment, optimize functioning and quality of life, extend community tenure, reduce use of unnecessary formal services and improve long-term care management [8, 9].

In 2005, the multinational consortium interRAI released the interRAI suite of Instruments [10]. The interRAI suite of instruments contains comprehensive geriatric assessment instruments, developed for different healthcare settings (interRAI Home Care, interRAI Acute Care, interRAI Long-term Care, interRAI Palliative Care, etc.) [11]. These instruments provide a comprehensive picture of the complex care problems of older people and allow the exchange of data between different care settings [12]. In Belgium, the interRAI-instruments were translated into Dutch, French and German during the BelRAI project [13, 14]. Furthermore, the instruments can be linked and filled out on a secured online web-application (belrai.org). Every interRAI-instrument includes a holistic questionnaire about the health condition of the patient and results that are calculated by means of internationally validated algorithms [15]. Based on these outcomes, care plans can be developed, evaluated and adjusted [15]. Examples of results are Scales, Quality Indicators (QIs) and Client Assessment Protocols (CAPs).

The interRAI Palliative Care instrument (interRAI PC) was developed as part of the interRAI suite of instruments [16]. To improve the continuity of care across settings, the interRAI PC is compatible with the other interRAI instruments [17]. The interRAI PC is a CGA that evaluates the diverse needs and preferences of adults nearing the end of life in all healthcare settings [16–18]. The aims of the instrument are to improve symptoms, enhance comfort, ameliorate quality of life and assist in coming to terms with death [18]. Outcomes of the interRAI PC are CAPs and Scales. The CAPs of the interRAI PC instrument are results that inform caregivers to which extent the client’s condition still can improve. The CAPs also inform whether improvement potential is lacking. The goals of care of different CAPs vary and contain the possibility to solve problems, to reduce decline or to create an atmosphere of improvement [13, 14]. The Scales of the interRAI PC are coherent calculations of client characteristics. These scales are conform to internationally validated scales (Depression Rating Scale, Cognitive Performance Scale, etc.) [13, 14].

The interRAI suite of instruments is tested and implemented in more than 30 countries and regions worldwide [10]. Reliability studies on the interRAI PC instrument have already been conducted in six countries [16, 17]. A systematic review also showed the interRAI PC instrument to be the most comprehensive geriatric assessment instrument that has been partially validated for nursing home residents with palliative care needs [19]. However, the review also identified that research on the effectiveness and a further validation of the instrument was needed [19].

This article describes the methods of a phase 0-I-II intervention that was conceived to best address our main research aims:

1. to evaluate the effect of the interRAI PC on the quality of palliative care in nursing homes;
2. to evaluate the feasibility of using the interRAI PC in nursing homes;
3. and to evaluate the face validity of the instrument (as research on the validation of the interRAI PC instrument in nursing homes is deficient).

The study has two additional research objectives:

4) to evaluate the predictive validity of the Palliative Index for Mortality (PIM) of the interRAI PC instrument (The PIM predicts the absolute and relative mortality risk within six months. If predictive validity is judged as high this index can internationally be used to detect the need for palliative care [20]);
5) to assess the convergent validity of the CAP ‘Mood’ of the interRAI PC instrument. (This CAP refers to depression, sadness and fear. These are psychological characteristics that regularly occur in persons with palliative care needs. These characteristics contribute to a diminished quality of life of the person and his or her family [21]).

The described study protocol will address the following specific research questions:

### Effect

Question 1. Does the use of the interRAI PC instrument improve the quality of spiritual, physical, psychosocial and emotional care for nursing home residents near the end of life?

### Feasibility

Question 2. Is the use of the interRAI PC instrument feasible for care professionals in nursing homes?

### Validity

Question 3. To what extent is the content of the interRAI PC experienced as complete, accurate and clear according to care professionals in nursing homes? (face validity).

Question 4. Is the Palliative Index for Mortality (PIM) of the interRAI PC instrument a good predictor of the risk of mortality within six months of nursing home residents? (predictive validity).

Question 5. Is the Client Assessment Protocol ‘Mood’ of the interRAI PC instrument a valid screening tool for depressive symptoms in nursing home residents with palliative care needs? (convergent validity).

## Methods

### Design of the complex intervention

**Figure 1:**
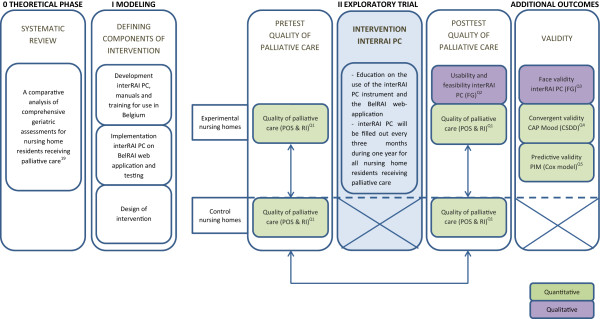
**Study model based on the MRC Framework for designing and evaluating complex interventions.**

This research covers phases 0, I and II of the Medical Research Council (MRC) framework for the design and evaluation of complex interventions (Figure 1) [22, 23]. The study has a longitudinal, quasi-experimental pretest-posttest design. Mixed methods of evaluation will be used. The intervention will be implemented in fifteen Belgian nursing homes. The multidisciplinary nursing home staff will fill out the interRAI PC for all nursing home residents aged 65 or older with palliative care needs.

#### Phase 0: Preclinical or theoretical

A systematic review was conducted to provide an overview of all existing CGAs that have been validated for nursing home residents with palliative care needs [19]. Psychometric properties of five identified CGAs have been appraised and their content was evaluated by means of the 13 domains for a palliative approach in residential aged care of the Australian Government Department of Health and Ageing (AGDHA) [19, 24].

Based on this systematic review, we found that the interRAI PC is the most comprehensive geriatric assessment to evaluate the palliative care needs of nursing home residents. The review also shows that future research should further examine the psychometric properties of the interRAI PC for use in nursing homes [19].

In the current study, we will take these findings further into practice to evaluate the effect, the feasibility and the validity of the interRAI PC instrument.

#### Phase I: Modeling

During the Modeling phase, the intervention is designed and decisions on what is measured or assessed are made [22].

The interRAI PC instrument, the manual and the CAPs guidelines were adjusted to two Belgian languages (Dutch and French). Furthermore, the instrument was implemented on the BelRAI web-application (belrai.org).

Additionally, the intervention was designed (Table 1), taking into account barriers and facilitators of implementing the interRAI-instruments in nursing homes, which have been identified during the BelRAI project [13, 14]. The main goal of the BelRAI project was to test the interRAI suite of instruments and the BelRAI webapplication in home care, acute care and residential care settings. Barriers and facilitators of implementing the interRAI-instruments in nursing homes have been identified, based on evaluation questionnaires and focus groups with the nursing home staff [13, 14].

**Table 1:** **Description of the intervention**

Preparatory phase		
Steps	Main objective	Secondary objective
Step 0	Introductory information	1. Introduction to the course of the project
		2. Introduction to the interRAI PC instrument
		3. Introduction to the BelRAI webapplication
		4. Identification of the pilot site (avalaibility of computers, laptops, tablets, internet,…)
		5. Capturing important dates (trainings, focus groups,…)
		6. Designation of a contact person
Step 1	Training on the interRAI PC and the BelRAI webapplication	1. One-day training on the interRAI PC and the use of the BelRAI webapplication
		2. Going through the course of the project (planning, questionnaires,…)
Step 2	Introduction on the interRAI PC and the BelRAI webapplication in the organization	1. Establishment of a work group
		2. Introduction of care professionals in the interRAI PC and the use of BelRAI
**Implementation phase**		
**Steps**	**Main objective**	**Secondary objectives**
Step 3	Identification of residents with palliative care needs, based on the surprise question	1. Identification of residents with palliative care needs, based on the surprise question
Step 4	Informed consent signature requirements	1. Designation of a person who is responsible for the informed consents
		2. The assigned responsible explains the project to the residents with palliative care needs or their representatives
		3. Informed consents are signed by the residents with palliative care needs or their representatives
Step 5	Login to the BelRAI webapplication	1. Care professionals apply for acces to the BelRAI webapplication by sending a written solemn declaration to the National Institute for Health and Disability Insurance (NIHDI)
		2. Care professionals login to BelRAI
Step 6	Definitions of roles and functions	1. Based on the profession and diploma of the caregivers, roles and functions are defined in the BelRAI system
		2. The group manager creates a group (the organization or the palliative department) in the BelRAI System
		3. The group manager ads client managers to the group
		4. Client managers create clients in the BelRAI system
		5. Client managers determine who has access to the clients
		6. The assessment manager closes the assessment
Step 7	Completion of the interRAI PC	1. Filling out the interRAI PC for all nursing home residents with palliative care needs
		2. Filling out the interRAI PC on a regular base (every three months)
		3. Multidisciplinary completion of the interRAI PC
Step 8	Interpretation of results	1. Care professionals discuss CAPs and Scales in team/with the patient/with the family
		2. Results are discussed every three months during one year
Step 9	Use of results	1. Results are used to develop a care plan or to adapt an existing care plan

As the introduction of the interRAI PC in clinical practice requires several steps at different stages, a training package for the care professionals of multiple disciplines was developed in order to support the user process of the interRAI PC and the BelRAI web-application.

#### Phase II: Exploratory trial

**General procedures** The effect of the interRAI PC on the quality of palliative care is evaluated, using a pretest-posttest design with quasi-random assignment to the intervention or control nursing homes. Control nursing homes provide care as usual.

**Setting and participants** Calls for participation are sent out by four umbrella organizations of Flemish nursing homes. An announcement is also made during a national conference for nursing home staff.

Fifteen volunteering nursing homes participate in the study. In the course of one year, these participating nursing homes will fill out the interRAI PC every three months for all residents aged 65 years or older with palliative care needs. The need for palliative care is evaluated by means of the surprise question (‘Would you be surprised if this person was to die within 6 to 12 months?’) [25, 26]. Based on a list of Flemish nursing homes from the National Institute for Health and Disability Insurance (NIHDI), fifteen care homes which match the intervention nursing homes regarding to facility size, ownership and geographic region are contacted for participation in a control group.

**Intervention** The fifteen experimental nursing homes receive the intervention that is being tested. The intervention consists of 11 steps. The complete intervention is described in Table 1. The fifteen control nursing homes provide care as usual and therefore do not receive the intervention.

**Sample size** Each year, on average 18 percent of the residents in a nursing home in Flanders, Belgium die [27]. Based on these data, this study aims at including at least 44 nursing home residents with palliative care needs, in order to detect an effect of the use of the interRAI PC instrument on the identified factors. Calculations are based on a medium effect size of 0.50, a two-sided significance level of 0.05 and a power of 0.90 (using G*Power 3.1.5 software) [28].

### Outcome measures

#### The effect of the interRAI PC instrument on the quality of palliative care

The multinational consortium interRAI has developed the interRAI PC instrument in order to improve palliative care for elderly [17]. In order to test this in practice, the effect of the interRAI PC on the quality of palliative care is evaluated.

**Psychometrics** An effect or impact evaluation assesses changes that can be attributed to a particular intervention, such as a project, program or policy, both the intended ones, as well as ideally the unintended ones [29].

**Data collection** Before the intervention period starts, both experimental and control nursing homes fill out the Palliative care Outcome Scale (POS) for all nursing home residents with palliative care needs. The POS is a ten-item outcome measure that assesses the quality of care in palliative patients [30]. The instrument contains items on physical, psychosocial and spiritual aspects. There are two versions of the instrument: the POS-Staff version and the POS-Patient version. In this study, we will use the POS-Staff version. The POS can also be used to assess persons with moderate to severe dementia [31]. The POS is a multidimensional scale that shows good psychometric properties. By means of the POS, the quality of care can be evaluated [32].

In addition, the experimental and control nursing homes fill out a Retrospective Information (RI) document. This document will provide data on the number of deaths in the past year, the place of death and the availability of a specific palliative care record.

After one year, both experimental and control nursing homes fill out the Palliative care Outcome Scale (POS) and the Retrospective information (RI) document for all nursing home residents with palliative care needs.

**Analysis** Quantitative data are analyzed with SPSS, using independent analysis of covariance (ANCOVA). Research has shown that ANCOVA with pretest-posttest data is the most appropriate statistical procedure in quasi-randomized control-group designs [33].

#### Feasibility of the interRAI PC instrument

Research has shown that an intervention, in order to be worthy of testing for efficacy, should answer the relevant questions within feasibility: ‘Can it work?’, ‘Does it work?’, ‘Will it work?’ [34].

**Psychometrics** Feasibility is defined as the convenience and suitability to use an instrument [35].

**Data collection** Focus groups and interviews with the multidisciplinary nursing home staff of participating nursing homes are organized. Caregivers are asked to answer questions concerning the length of the instrument, barriers, facilitators, completion time, planning, etc.

**Analysis** Qualitative data analysis will be conducted using the NVivo software©.

#### Face validity of the interRAI PC instrument

Based on a systematic review, we found that the interRAI PC instrument is the most comprehensive geriatric assessment for nursing home residents with palliative care needs [19]. In order to take this finding further into practice, the face validity of the interRAI PC will be evaluated.

**Psychometrics** Face validity refers to the degree to which an instrument appears to reflect the variable it has been designed to measure [36].

**Data collection** Focus groups and interviews with multidisciplinary nursing home staff of participating nursing homes are organized to evaluate the face validity of the instrument. Caregivers are asked whether items, CAPs and scales are lacking, redundant and clear enough.

**Analysis** Qualitative data analysis will be conducted using the NVivo software©.

#### Predictive validity of the palliative index for mortality (PIM) of the interRAI PC instrument

Based on risk factors, the PIM of the interRAI PC instrument predicts the absolute and relative mortality risk within six months. This index can be used to detect the need for palliative care [20].

**Psychometrics** Predictive validity is defined as ‘the extent to which a score on a scale or test predicts scores on some criterion measure’ [37].

**Data collection** Caregivers fill out the PIM of the interRAI PC instrument every three months during one year for all residents with palliative care needs as identified through the surprise question.

**Analysis** A survival analysis will be conducted using the Cox proportional hazards regression model. A survival analysis deals with time until the occurrence of a particular event [38, 39]. In this study, the event of interest is death of the resident. The Cox model is a widely used method for survival analysis in examining time-to-event data [40]. One of the advantages of the Cox model is that it maintains the variable in its original quantitative form [40]. Based on previous research on the mortality prediction of the Mimimum Data Set Mortality Risk Index (former PIM), a concordance statistic of 0.75 will be considered as indicative for good predictive value [20]. Statistical data analysis will be conducted using SPSS.

#### Convergent validity of the client assessment protocol ‘Mood’ (CAP ‘Mood’)

The CAP ‘Mood’ of the interRAI PC instrument refers to depression, sadness and fear. These are psychological characteristics that regularly occur in persons with palliative care needs. These characteristics contribute to a diminished quality of life of the person and his or her family [21].

**Psychometrics** Convergent validity is a subtype of construct validity and is defined as the degree to which two measures of constructs that theoretically should be related, are in fact related [41].

### Data collection

The CAP Mood is tested against a ‘gold standard’ in order to answer the second research question. The Cornell Scale for Depression in Dementia (CSDD) is used as the ‘gold standard’. Together with the completion of the interRAI PC instrument, an independent assessor also completes the CSDD every three months during one year for all residents with palliative care needs. The CSDD is a 19-item scale, designed for the screening of depressive symptoms in older adults with and without dementia. The CSDD is the only depression scale that has been validated for both populations [42, 43]. Research shows that the instrument is equally valid in populations with or without dementia [44]. In prior research, the CSDD already has been used as a ‘gold standard’ [45].

**Analysis** The level of agreement between the CAP Mood and the CSDD test scores will be explored using comparisons of means, agreement coefficients and diagnostic accuracy. A CSDD cut-off of 8 will be used as ‘gold standard’. Probability levels of 0.05 will be considered significant [45]. Statistical data analysis will be conducted using SPSS.

### Ethics statement

Approval to conduct this research was granted by the Belgian Commission for the Protection of Privacy (BCPP) and the UZ Leuven Medical Ethics Committee (file number B322201421986).

All nursing home residents with palliative care needs or their representatives are asked to sign an informed consent agreement. Refusing to participate will not affect the care services offered to the resident. When residents or their representatives decide to participate, they can withdraw their consent at any time.

A formal procedure was undertaken to enable caregivers to fill out the interRAI PC on a secured online web-application (belrai.org).

## Discussion

This article describes the research protocol of a mixed methods study with a longitudinal, quasi-experimental pretest-posttest design. The study is based on phases 0, I and II of the Medical Research Council (MRC) Framework [22, 23]. The main aims of the study are to evaluate (1) the effect of the interRAI PC on the quality of palliative care in nursing homes; (2) the feasibility of the interRAI PC; (3) the face validity of the interRAI PC; (4) the predictive validity of the Palliative Index for Mortality (PIM) of the interRAI PC; (5) and the convergent validity of the CAP ‘Mood’ of the interRAI PC.

Previous research has shown a need for well-validated CGAs in clinical practice to evaluate an older person’s medical, psychosocial and functional condition [46]. Based on a systematic review, the authors found the interRAI PC to be the most comprehensive geriatric assessment for palliative care in nursing homes. However, insufficient data are available on the validity and the effectiveness of the instrument in a nursing home population [19]. Therefore, this research is conducted on the effect, the feasibility and the validity of the interRAI PC instrument in a population of nursing home residents with palliative care needs.

The interRAI PC consists of various components and the introduction of the interRAI PC in clinical practice requires several steps at different stages [47]. Interventions ‘comprising a number of separate elements which seem essential to the proper functioning of the interventions although the ‘active ingredient’ of the intervention that is effective is difficult to specify’ are considered as complex interventions [48]. For trials of such complex interventions, Campbell et al. recommended the MRC framework [22, 23].

The phased MRC approach, which combines qualitative and quantitative methods, improves the study design and implementation and will contribute to a higher generalizability of the results [23]. A supplementary strength of the study lies in the fact that it is longitudinal. Several observations of the same variables will be conducted over a period of time. In addition, changes, developments and learning processes over time can be detected. Because of the pretest-posttest design and the use of a control group, expected improvements in the quality of palliative care can be examined.

Limitations of the study also need to be acknowledged. Because of time restrictions, the interRAI PC instrument will only be filled out during one year. Therefore, the maximum amount of longitudinal measures is limited to four. Furthermore, the experimental group consists of all nursing home residents with palliative care needs of fifteen nursing homes (public, private, profit and private commercial nursing homes). However, this sample might not be representative of the general Flemish nursing home population. Additionally, due to ethical and practical constraints, participants will not be randomly assigned to the intervention or control group. Because of the strong commitment requirements, nursing homes which volunteer in the experimental group are all included. Moreover, it is impossible to refuse caregivers to fill out the interRAI PC since the instrument is made accessible through the online webapplication BelRAI. Nursing homes are thus included in the experimental group if they are interested in filling out the interRAI PC.

The final result of the study will be a psychometrically evaluated CGA for nursing home residents with palliative care needs, with outcomes that can be used for care planning. If the validity and the effect of the use of the interRAI PC instrument are evaluated positive, this study protocol can form the basis for a larger-scale phase III randomized controlled trial and a broader implementation of the interRAI PC in nursing homes (phase IV). This can further enhance and stimulate optimal palliative care for nursing home residents and eventually also for adults living in other settings. Finally, the international relevance of this study protocol needs to be acknowledged. While the interRAI suite of instruments -and more specifically the interRAI PC instrument- is tested and implemented worldwide, this study protocol can provide guidelines to evaluate the effect, the validity and the usability of interRAI PC in other countries.

## Authors’ information

1. KH – MSPsy, PhD Student*
2. NS – MSPsy, PhD, Postdoctoral researcher*
3. JC – MSSoc, PhD, Professor^Ɨ^
4. CVA – MSPsy, PhD, Professor*
5. AD – MSSoc, PhD, Professor*

*KU Leuven, LUCAS, Center for Healthcare and Consultancy, Kapucijnenvoer 39/5310 B-3000 Leuven, BELGIUM

^Ɨ^Vrije Universiteit Brussel (VUB) & Ghent University, Laarbeeklaan 103 B-1090 Brussels, BELGIUM.

## Abbreviations

CGAs: Comprehensive geriatric assessments
interRAI PC: interRAI palliative care instrument
CAPs: Client assessment protocols
MRC: Medical research council
PIM: Palliative index for mortality
QIs: Quality indicators
AGDHA: Australian government department of health and ageing
NIHDI: National institute for health and disability insurance
POS: Palliative care outcome scale
RI: Retrospective information
ANCOVA: Analysis of covariance
CSDD: Cornell scale for depression in dementia
BCPP: Belgian commission for the protection of privacy.
